# 2nd-order random lasing in a multimode diode-pumped graded-index fiber

**DOI:** 10.1038/s41598-018-35767-9

**Published:** 2018-11-30

**Authors:** Ekaterina A. Evmenova, Alexey G. Kuznetsov, Ilya N. Nemov, Alexey A. Wolf, Alexandr V. Dostovalov, Sergey I. Kablukov, Sergey A. Babin

**Affiliations:** 1 Institute of Automation and Electrometry SB RAS, Novosibirsk, 630090 Russia; 2 Novosibirsk State University, Novosibirsk, 630090 Russia

## Abstract

Raman lasing in a graded-index fiber (GIF) attracts now great deal of attention due to the opportunity to convert high-power multimode laser diode radiation into the Stokes wave with beam quality improvement based on the Raman clean-up effect. Here we report on the cascaded Raman generation of the 2nd Stokes order in the 1.1-km long GIF with 100-μm core directly pumped by 915-nm diodes. In the studied all-fiber scheme, the 1st Stokes order is generated at 950–954 nm in a linear cavity formed at GIF ends by two fiber Bragg gratings (FBGs) securing beam quality improvement from M^2^ ≈ 30 to M^2^ ≈ 2.3 due to special transverse structure of FBGs. The 2nd Stokes wave is generated either in linear (two FBGs) or half-open (one FBG) cavity with random distributed feedback via Rayleigh backscattering. Their comparison shows that the random lasing provides better beam quality and higher slope efficiency. Nearly diffraction limited beam (M^2^ ≈ 1.6) with power up to 27 W at maximum gain (996 nm), and 17 W at the detuned wavelength of 978 nm has been obtained, thus demonstrating that the 2^nd^-order random lasing in diode-pumped GIF with FBGs provides high-efficiency high-quality beam generation in a broad wavelength range within the Raman gain spectral profile.

## Introduction

Outstanding performance of high-power fiber lasers in both CW and pulsed regimes^1–3^ enables their wide implementation for material processing in automotive, aerospace and oil & gas industries, and for micromachining in microelectronics, photovoltaics, LED, medical devices etc. The highest CW power is achieved for Yb-doped fiber laser (YDFL) amounting to 10 kW at ~1.07 μm in single-transverse-mode (singlemode) regime^4,5^. This became possible due to the implementation of key fiber laser technologies such as fiber-based pump combining, cladding pumping of singlemode Yb-doped fiber and its splitting into two stages (so called tandem pumping) when laser diodes (LDs) pump YDFLs which then pump another YDFL at the second stage, see^1,2^ for a review. Output power up to 70 kW is shown to be feasible with the tandem pumping^6^, but the development of mode instability and stimulated Raman scattering (SRS) set further limits on stability and power of such lasers^1,2^.

At the same time, this negative feature of SRS may be converted into a positive effect if one uses it for achieving Raman lasing at new wavelengths, which are not available from YDFLs and other rare-earth (RE) doped fiber lasers. Raman fiber lasers (RFLs) usually utilize singlemode passive fibers^7^ pumped into the core by singlemode RE-doped fiber lasers. Output power of RFLs pumped by high-power YDFLs exceeds 100 Watt level for the first^8^ as well as for higher Stokes orders^9–11^. In the latter case, cascaded generation of intermediate and output Stokes waves is supported by the nested cavities made of fiber Bragg grating (FBG) pairs^9–11^, either in a single laser or in a master oscillator - power amplifier (MOPA) configurations based on passive fibers. Power scaling to kW level is demonstrated for an integrated Yb-Raman fiber amplifier, in which both amplification and Raman conversion occur within the Yb-doped fiber MOPA laser^12,13^. A much simpler RFL architecture (without nested FBG cavities or MOPA schemes) utilizes Rayleigh backscattering in a fiber itself for broadband random distributed feedback^14^. Such random Raman fiber laser (RRFL) provides high-efficiency generation of the first or higher Stokes orders by increasing pump power, see^15^ and citation therein. Power scaling to 418 W appears to be possible in a RRFL based on LMA passive fibers^16^. Note that all the discussed RFL schemes use for pumping a high-power singlemode YDFL, which in its turn is cladding pumped by high-power multimode LDs.

Recently, Raman lasers based on a graded-index fiber (GIF) attract great deal of attention due to the opportunity of its direct pumping by high-power laser diodes^17–20^. As a GIF is highly multimode, multimode LD radiation is efficiently coupled into the large fiber core. At the same time, quality of the generated beam may be greatly improved compared to the pump one due to the well-known Raman beam clean-up effect^21^. Using commercially available GIFs and 9xx-nm LDs, such RFLs may generate at wavelengths <1 μm, where high-power generation of RE-doped fiber lasers is hardly possible. The first CW LD-pumped RFL operating below 1 μm has been demonstrated in^17^: for a 4.5-km-long GIF with 62.5-μm core, the output beam with power of ~3 W at 980 nm has exhibited much better quality than the LD pump beam at ~940 nm. The possibility to scale output power of LD-pumped RFLs up to 20 W^18^, 80 W^19^ and 154 W^20^ was demonstrated using shorter (1.5, 0.5 and 0.2 km, respectively) 62.5-μm GIFs and higher pump powers of 976-nm LD modules providing Raman output at ~1020 nm, i.e. in the spectral range of YDFLs. In spite of significant improvement of the beam quality, the Raman generation obtained with bulk mirrors remains to be far from singlemode one: M^2^ = 4–8 while using conventional pump LDs with M^2^ ~ 20^18,20^. With 915-nm LDs, Raman generation at 954 nm has been obtained^22^. The beam quality becomes near diffraction-limited (M^2^ ~ 1.3) at ~10 W power obtained in the cavity made of special fiber Bragg gratings^23^: the fundamental mode selection is provided here by the output FBG inscribed by femtosecond (fs) pulses in the near-axis area of 62.5-μm GIF cross section. As a next step, coupling of several LDs through a multimode fiber pump combiner allows one to increase the coupled pump power and to develop an all-fiber structure of the multimode Raman laser^24^. The all-fiber configuration has obvious advantages such as scalability, compactness, reliability, and long-term stability. Output power at 954 nm increases to 49 W with the fiber core enlargement to 85 μm, at the expense of slight beam quality reduction to M^2^ ≈ 2.6. A further core enlargement to 100 μm in the same configuration (including the same LDs) results in the enhancement of output power up to 62 W and slope efficiency up to 84%, see^25^ for details of FBG-based GIF Raman lasers with LD pumping. Let us note that an attempt to develop random RFL based on Rayleigh backscattering in 62.5-μm GIF with direct LD pumping through bulk optics^26^ has demonstrated a rather low output power (<1 W at 980 nm).

Here we report on the first successful endeavor of developing a high-power LD-pumped random RFL. We explore an opportunity of cascaded generation at the 2nd Stokes order in the 1.1-km long 100-μm core GIF directly pumped by 915-nm LDs in an all-fiber configuration. Coupling of several LDs to the GIF is provided by a special multimode fiber pump combiner^24,25^, a linear cavity formed by special fiber Bragg gratings (FBGs) secures the 1st Stokes wave (~954 nm) generation with relatively high beam quality (M^2^ ≈ 2.3 at 35 W) at low quality of the LD pump beam (M^2^ ≈ 30), see^27^ for details. It has been shown that the 2nd Stokes wave generated in a half-open cavity with one FBG and random distributed feedback based on the Rayleigh backscattering in the multimode GIF exhibits nearly diffraction limited output beam (M^2^ ≈ 1.6). Output power as high as 27 and 17 W has been obtained at the wavelengths of 996 and 978 nm, corresponding to the Raman gain maximum and two thirds of the Stokes shift, respectively. The obtained results demonstrate that the random lasing in a LD-pumped GIF provides high-quality high-efficiency generation in a broad wavelength range within the Raman gain spectral profile.

## Results and Discussion

### Experiment

**Figure 1:**
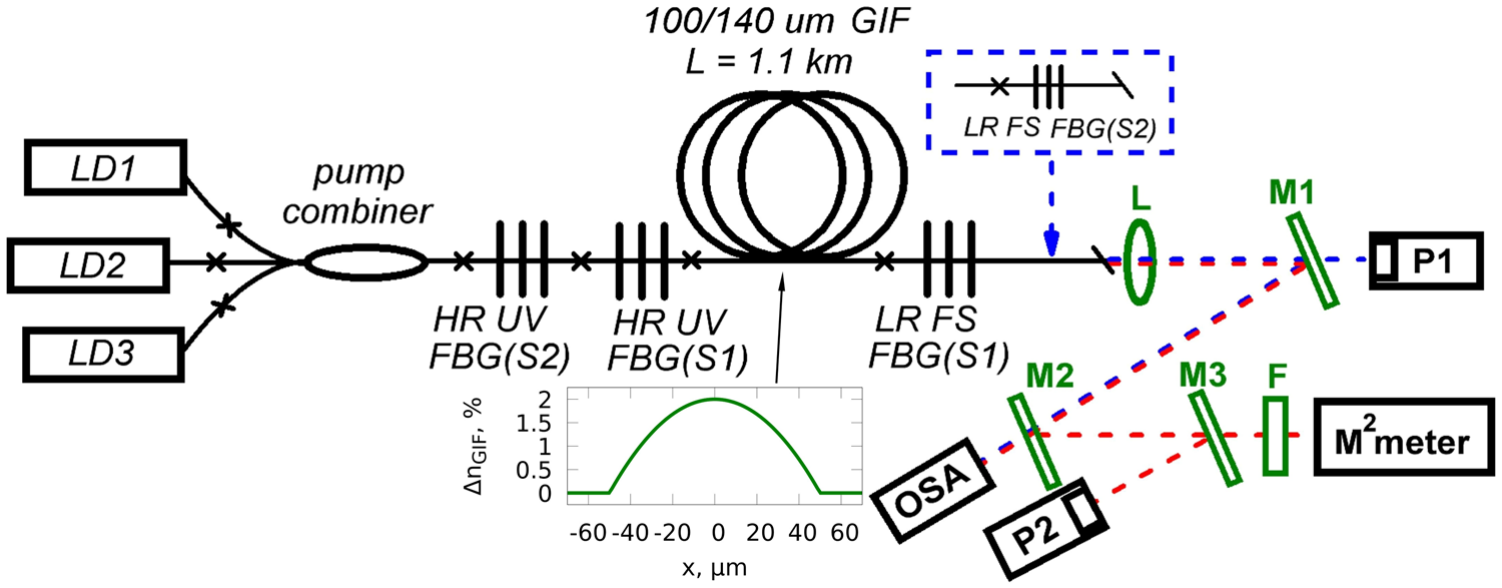
All-fiber configuration of the cascaded random RFL with direct LD pumping: LD1, LD2, LD3 – multimode laser diodes; HR UV FBG – high-reflection fiber Bragg grating inscribed by UV radiation; LR FS FBG – low-reflection fiber Bragg grating inscribed by femtosecond (FS) pulses; L – collimating lens; M1, M2, M3 – dichroic mirrors; F – bandpass filter; P1, P2 – power meters; OSA – optical spectrum analyzer. S1 and S2 designate FBGs for the first and the second Stokes component, respectively. When the LR FS FBG(S2) is added in the scheme, RRFL becomes a conventional RFL at the second Stokes wavelength.

Experimental scheme of the cascaded RFL based on the 100-μm core GIF is presented in Fig. 1. Pump radiation from three fiber pigtailed high-power LDs operating at the wavelength of 915 nm is added together by a 3 × 1 multimode fiber pump combiner. Core diameter and numerical aperture of the LD pigtail fibers are 105 μm and 0.15–0.22, correspondingly. The input ports of the fused pump combiner are made of multimode step-index fiber with 105-μm core and output port is made of a 100-μm core GIF with numerical aperture of 0.29. It is spliced to the same 1.1-km-long 100-μm core GIF with the Raman gain provided by LD pumping. The pump coupling losses including those at the pump combiner and splice points amount to about 25%. The linear laser cavity for the 1st Stokes wave at wavelength of 950 nm or 954 nm is formed in the GIF by a high-reflection UV FBG and an output low-reflective FS FBG (both at the corresponding 1st Stokes wavelength S1). In case of conventional cascaded RFL scheme, a cavity for the 2nd Stokes order at wavelength of 976 nm is also formed by two FBGs: HR UV FBG(S2) and LR FS FBG(S2). The reflectivity of input and output FBGs is ~90% and ~4%, respectively. It should be noted that the HR FBG is inscribed by CW UV interference pattern formed in the core area, while the LR FBG is inscribed point-by-point by femtosecond pulses tightly focused in the center of the GIF core thus providing the selection of fundamental transverse mode^23^.

A half-open cavity with random distributed feedback is implemented by one HR UV FBG(S2) at 978 nm or 996 nm wavelength. The output fiber end is cleaved in this case with an angle of >10° to eliminate Fresnel reflection. Therefore, the feedback is provided by the reflection from HR FBG at one GIF end and the Rayleigh backscattering distributed along the GIF. Dichroic mirrors M1, M2, M3 are used to separate pump radiation at 915 nm and cascaded Raman generation at >950 nm. Major part of their power (95%) gets on power meters P1 and P2, respectively, while the residual radiation passed through mirrors M2 and M3 is used to measure output spectrum and profile of the generated beam by an optical spectrum analyzer (OSA) and Thorlabs M^2^-measurement system, respectively. A set of bandpass filters F with different central wavelength is used to measure quality parameter M^2^ of the 1st or the 2nd Stokes beams. In the case of conventional cascaded RFL, the 1st and the 2nd Stokes radiations are separated by additional dichroic mirrors and their output powers are measured directly. In the case of random RFL, output powers of different Stokes orders are calculated using relative power dependences measured by the OSA for the individual Stokes emission lines and the total power measured by power meter P2.

As a first step, we have compared conventional and random Raman lasing of the 2nd Stokes order at 976–978 nm. Then we have compared random lasing at different 2nd Stokes wavelengths (978 and 996 nm) with a slight variation of the 1st Stokes wavelength (950 and 954 nm) in addition.

### Comparison of conventional and random RFLs operating at ~976 nm

**Figure 2:**
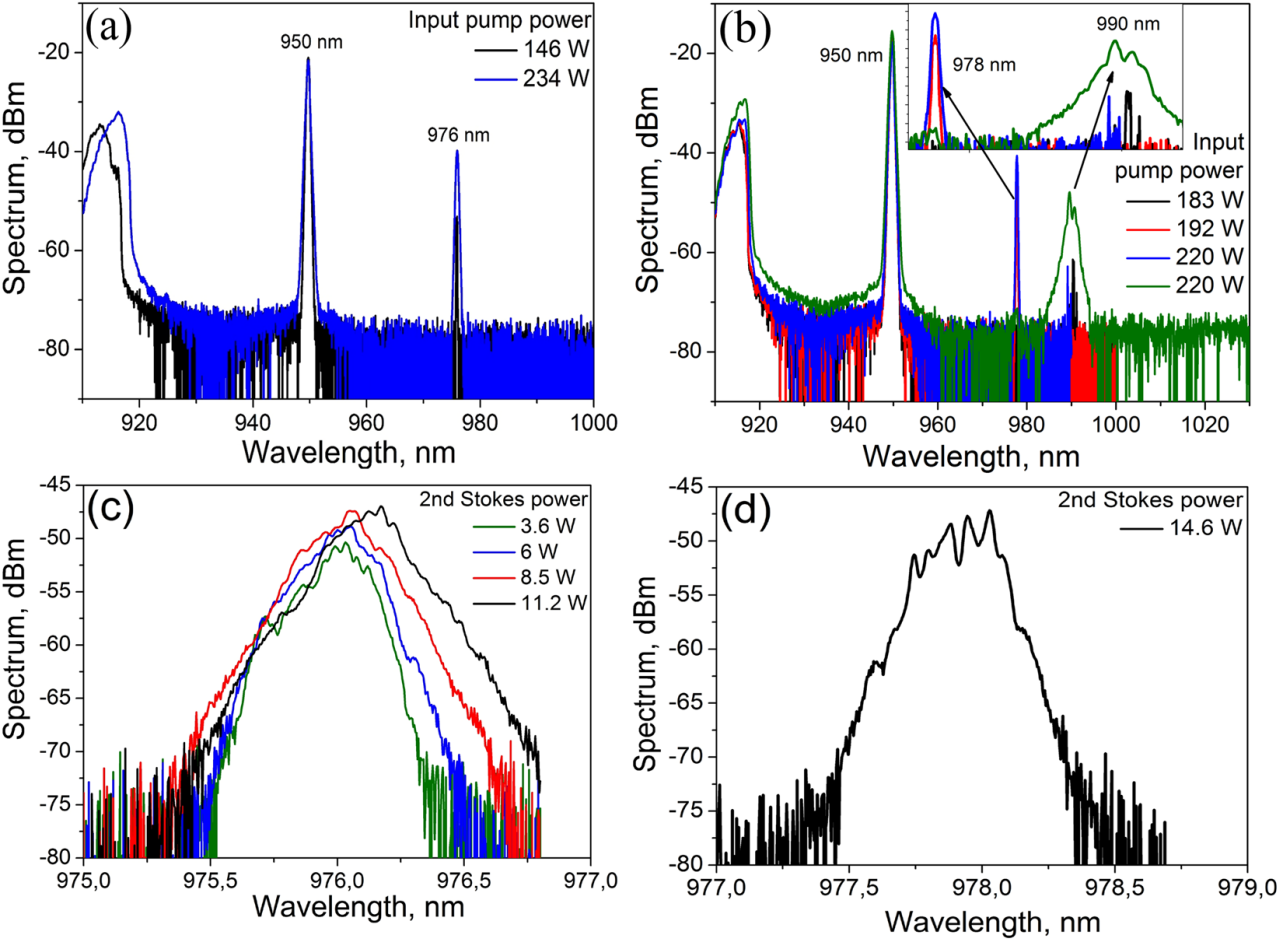
Output spectrum of conventional (**a**) and random (**b**) RFL with the first and the second Stokes wavelengths of 950 nm and 976–978 nm, respectively, as dependent of the LDs pump power launched into the GIF, zoomed spectrum in (**b**) demonstrates appearance of stochastic generation at 990 nm. Corresponding zoomed spectra at the second Stokes wavelength in the conventional (**c**) and random (**d**) RFL configurations.

Let us first compare the cascaded Raman generation in multimode 100-µm GIF with conventional RFL and random RFL cavity configurations. For the comparison, the 1st Stokes wavelength was set at 950 nm and the 2nd Stokes wavelength was set at 976 nm (RFL) or 978 nm (RRFL) by means of corresponding FBGs (see Fig. 1). The generation wavelength near 976 nm was chosen because of its wide use in pumping schemes of YDFLs. The Raman frequency shift for the 1st Stokes wave in this scheme amounts to ~410 cm^−1^ that is close to the maximum of the Raman gain in a highly Ge-doped silica fiber (420–440 cm^−1^ depending on the GeO_2_ concentration^28^), while the shift for the 2nd Stokes wave ~300 cm^−1^ is far from the gain maximum. Figure 2(a) and (b) show the output spectrum in RFL and RRFL configurations, respectively, at different input pump powers. In the case of RFL, generation at two wavelengths only (950 nm and 976 nm) is observed. In the case of random RFL, relatively weak stochastic (time-to-time) generation at ~990 nm is also seen. This wavelength corresponds to the maximum of the 2nd-order Raman gain. Near the threshold the maximum Raman gain dominates over the FBG reflection. Then the generation moves to the FBG wavelength (978 nm) generated in a broad range of powers, whereas at maximum pump power (220 W) stochastic generation at 990 nm is also observed in addition to the main peak at 978 nm (two spectra measured at different moments are given).

Zoomed spectra of the 2nd Stokes wave measured with resolution of 0.02 nm for two laser configurations are presented in Fig. 2(c) and (d). For RFL configuration, they are relatively smooth at all powers with the −3 dB bandwidth of about 0.25 nm, whereas more pronounced fluctuations are seen near the line center for the RRFL configuration which change from scan to scan. At the same time, the envelope is close to hyperbolic secant shape of the width defined by HR FBG in both cases, in accordance with the turbulence-like broadening model applicable in both cases, see^29,30^ and citation therein.

**Figure 3:**
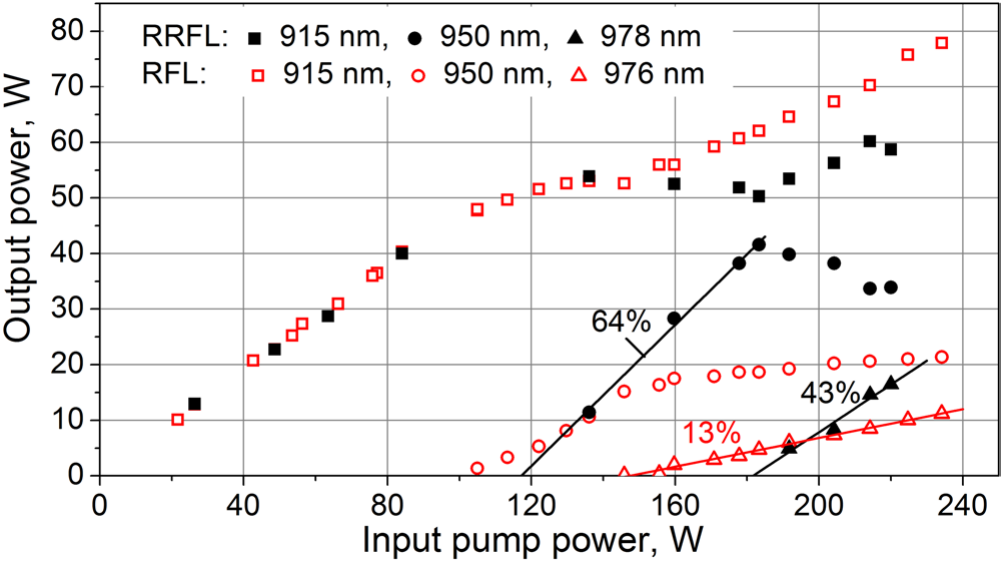
Measured output power of the random (filled symbols) and conventional (open symbols) RFL at the first Stokes wavelength of 950 nm (circles) and the second Stokes wavelength 976 or 978 nm for RFL and RRFL respectively (triangles) and transmitted pump power at 915 nm (squares),versus the input pump power.

Power characteristics of the conventional and random RFL configurations are compared in Fig. 3. It is seen that the 2nd Stokes threshold is higher for the random RFL: 180 W instead of 150 W for the conventional RFL, but the slope efficiency is also much higher: 43% instead of 13% for the conventional RFL. This behavior qualitatively agrees with the result obtained for multiwavelength RFL and RRFL configurations based on a singlemode fiber^31^. It is explained by much lower intensity (and narrower spectrum, as a result) of the incoming light at HR FBG in case of RRFL, whereas its spectral broadening (approaching the FBG width) in case of RFL leads to higher nonlinear losses at the light reflection by HR FBG. It should be noted that in multimode GIF the difference in efficiency of Raman lasing for half-open and conventional cavities is even more pronounced.

### Comparison of random RFLs operating at different wavelength within the Raman gain spectral profile

**Figure 4:**
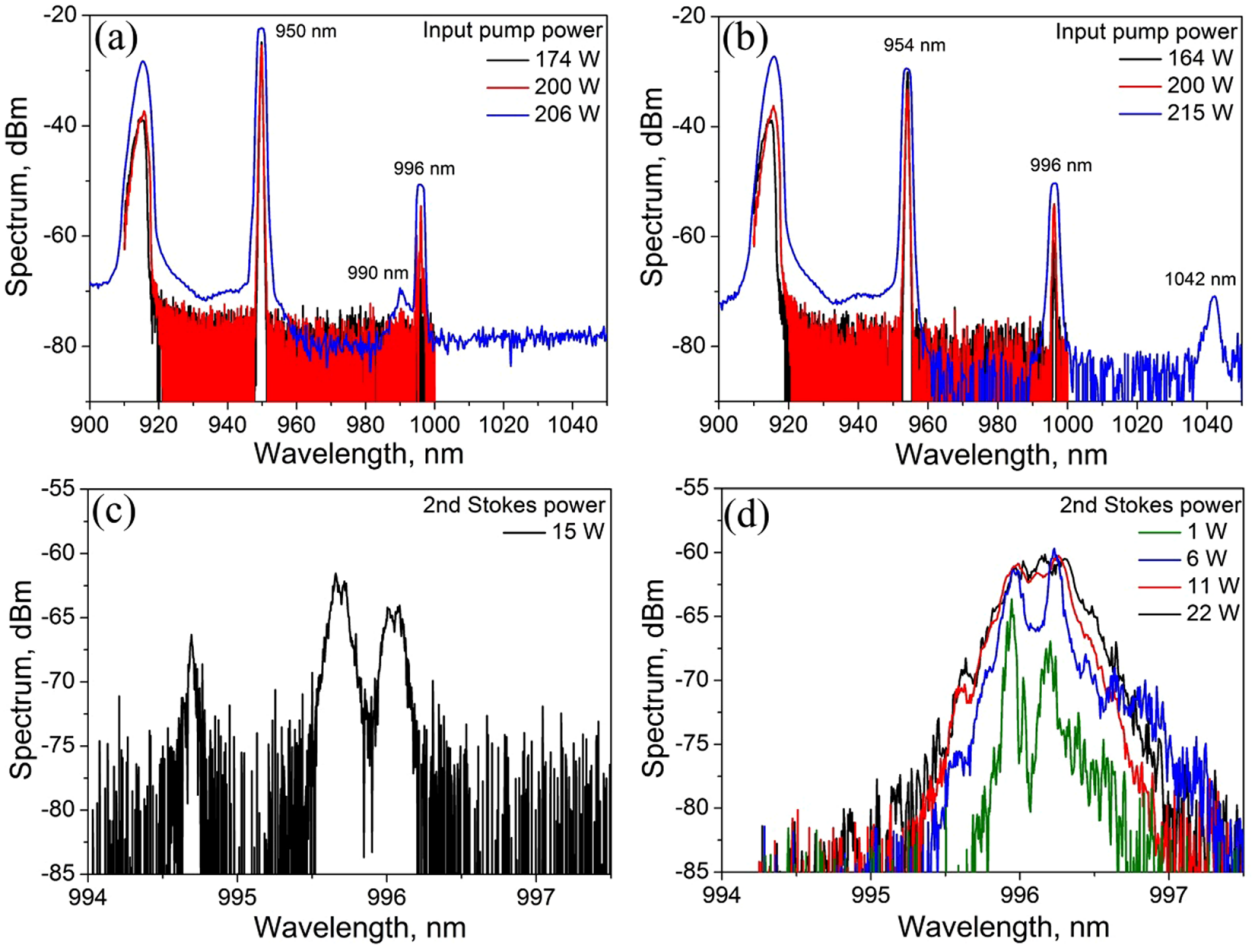
Output spectrum of the random RFL with the first Stokes wavelengths of 950 nm (**a**) or 954 nm (**b**) and the second Stokes wavelength of 996 nm at different LD pump power launched in the GIF. Corresponding spectra at the 2nd Stokes wavelength in the case of 950 nm (**c**) and 954 nm (**d**) at different second Stokes powers.

In order to compare 2^nd^-order RRFL generation at different wavelengths, FBG(S2) with reflection at 996 nm has been also tested. We have used two different wavelengths for the 1^st^ Stokes wave − 950 nm and 954 nm, thus varying Raman frequency shift in this configuration. So, we explore 954/996 nm generation with 2nd Stokes shift of 442 cm^−1^ nearly corresponding to the Raman gain maximum and 950/996 nm generation with 486 cm^−1^ shift corresponding to the long-wavelength slope of the Raman gain profile, and compare them to each other and to the case of 950/978 nm generation with 300 cm^−1^ shift described in previous paragraph. Figure 4(a) and (b) show the 2nd order RRFL spectra at different input pump power values for the 950/996 nm and 954/996 nm RRFL configurations, respectively. In the case of 1st Stokes generation at 950 nm we observe stochastic generation at 990 nm (corresponding to the maximum Raman gain in this case), but at the maximum pump power only. In the case of the maximum gain for both Stokes orders (954 and 996 nm), there are no additional emission lines, but at maximum power near-threshold generation of the 3rd-order Stokes wave at 1042 nm appears. Zoomed view of the 2nd Stokes spectrum shows that in the case of 486 cm^−1^ Raman shift (Fig. 4c) the spectrum contains additional blue-shifted lines corresponding to the resonant coupling of higher-order transverse modes of the GIF with HR FBG since they have higher Raman gain than the fundamental one. This result is similar to that one obtained for the conventional RFL pumped by multimode LDs when the shift corresponds to the long-wavelength slope of the Raman gain profile^27^. In the case of maximum Raman gain (Fig. 4d), the 2nd Stokes spectrum consists of two sharp peaks which merge into one broader peak with the bandwidth of ~0.5 nm at high powers. This is quite different from the case of conventional RFL (see Fig. 2c), which spectrum is always single-peaked with the bandwidth slightly increasing with power. This difference deserves an additional detailed study.

**Figure 5:**
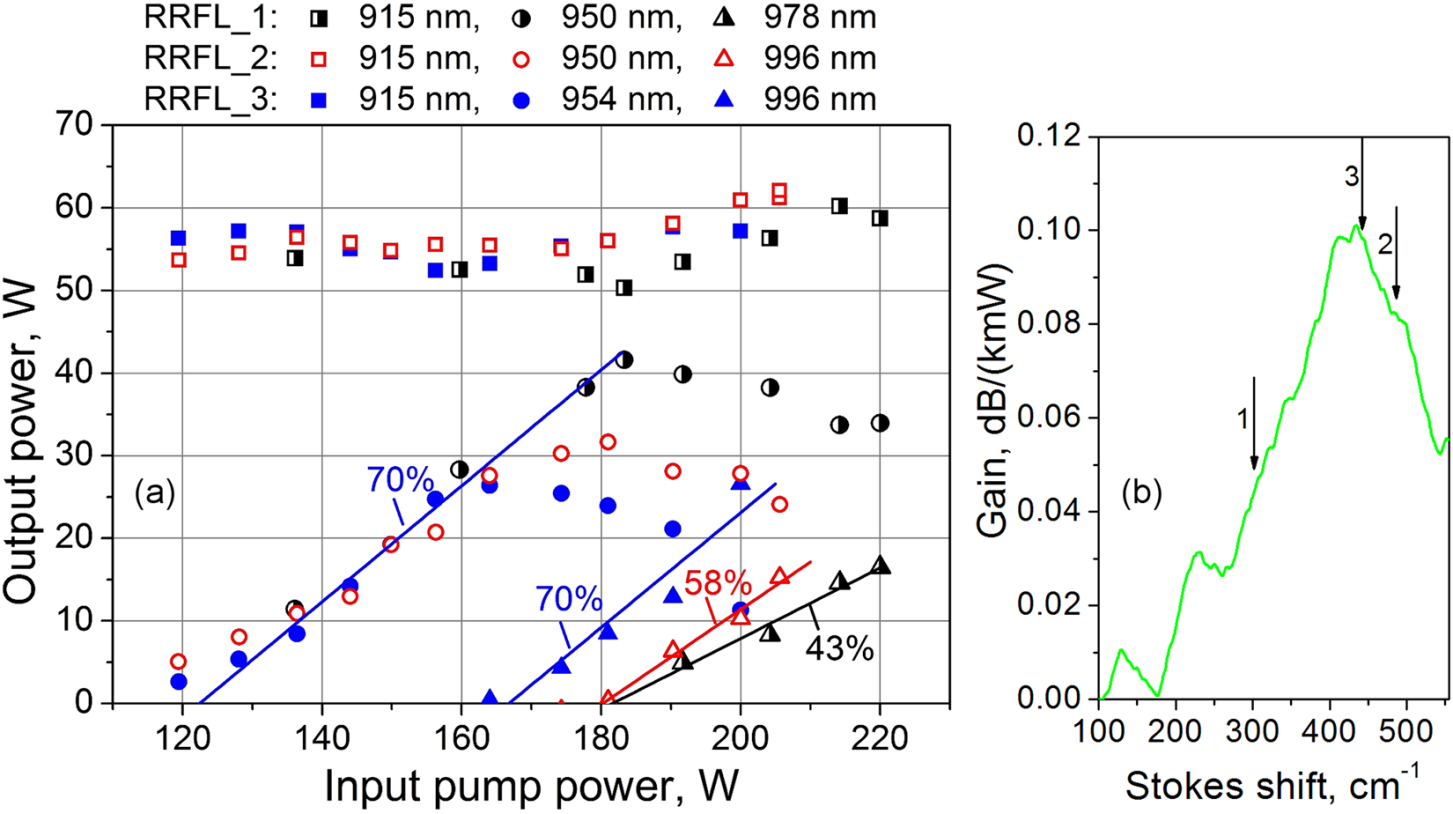
(**a**) Measured output power at the pump (squares), the first (circles) and the second (triangles) Stokes wavelength versus the input pump power for three different random RFL configurations with 2nd order Raman shift of 300 (RRFL 1), 486 (RRFL 2) and 442 (RRFL 3) cm^−1^. (**b**) Corresponding positions of the shift on the Raman gain profile.

Comparison of the 2nd Stokes power for three different RRFL configurations (Fig. 5a) shows that the closer Raman shift to the gain maximum (corresponding positions on the measured Raman gain profile^27^ are shown in Fig. 5b), the lower the threshold and the higher slope efficiency and maximum output power of the laser at available pumping. Thus, we have obtained maximum slope efficiency of 70% and the output power of 27 W at 2nd Stokes wavelength of 996 nm with 1st Stokes wavelength of 954 nm (RRFL3), which corresponds to the Raman gain maximum in the 1.1-km-long Random RFL based on 100-μm core GIF. Note that the transmitted pump power and the 1st Stokes power (before 2nd Stokes threshold) are almost the same for all configurations, whereas beyond the 2^nd^ Stokes threshold they diverge significantly but less than the difference between RRFL and RFL (see Fig. 3). The maximum slope efficiency of LD pump to 2nd Stokes wave conversion obtained in configuration RRFL3 is nearly the same as that for the pump to 1st Stokes wave conversion. Maximum optical power in this case is also limited by the threshold of the next Stokes order generation.

**Figure 6:**
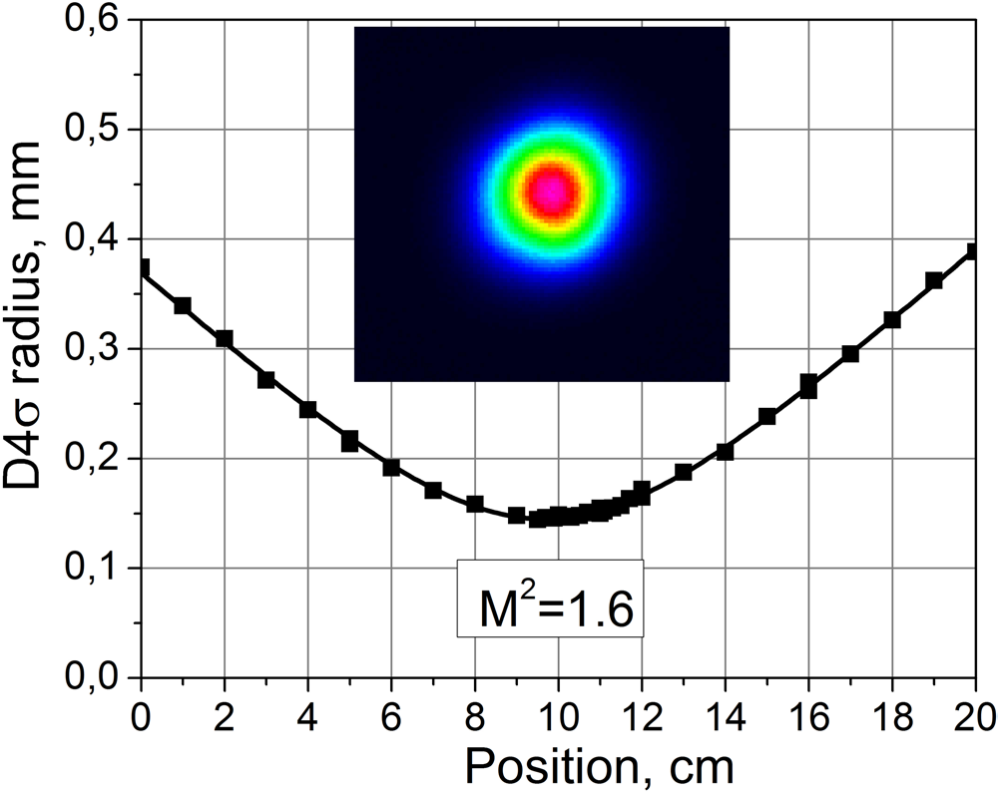
Parameter M^2^ measurements for the generated beam at 996 nm of the RRFL with 12.9 W output power. Inset: intensity profile of the generation in the waist.

Beam quality parameter M^2^ measured for 996-nm radiation is shown in Fig. 6. The measured value is nearly constant (~1.6) at powers up to 13 W and does not change significantly at higher powers that is confirmed by output beam profile monitoring at increasing power. This value is considerably lower than that for the 1st Stokes beam (~2.3). It is also lower than that for the 2nd Stokes generation in conventional RFL at 978 nm (1.9). Beam pattern of the RRFL output shown in the inset of Fig. 6 is close to the intensity profile of the fundamental mode. Thus, nearly diffraction limited beam is generated in the configuration with Rayleigh scattering based distributed feedback.

## Conclusion

Thus we have demonstrated for the first time high-power (27 W) 2^nd^-order random lasing at 996 nm in the all-fiber scheme of directly LD-pumped (at 915 nm) multimode graded-index fiber Raman laser with FBG cavity formed for the intermediate 1st order Stokes wave (954 nm) with high slope efficiency (~70%) of LD pump to 2^nd^ Stokes wave conversion. At that, nearly diffraction limited output beam (M^2^ ≈ 1.6 almost independent on power) is generated with the Rayleigh backscattering based random distributed feedback. The random generation in multimode GIF appears to be more efficient and provides higher beam quality than the cascaded generation in conventional RFL scheme with nested FBG cavities for all Stokes orders. So, the developed laser combines three types of beam cleaning effects: beam clean-up at Raman conversion^21^, low-order transverse mode selection by special FBGs at the intermediate stage^23,24^, and the influence of Rayleigh backscattering at the final (random) stage of cascaded Raman generation in the graded-index fiber. Increasing output power to the level of hundreds Watts may result in emergence of Kerr self-cleaning effect recently discovered in pulsed systems^32,33^.

We have also shown that such random laser is able to operate in a broad range of wavelengths within the Raman gain profile with relatively high efficiency and power: 17 W with 43% slope efficiency is obtained at the wavelength of 978 nm far detuned (~20 nm) from the Raman gain maximum. Next cascade (3^rd^-order Stokes) generation threshold is shown to be achievable in the presented scheme due to the high quality of the generated beam.

In conclusion, we offer high-brightness laser source of new type based on cascaded random lasing in a simple configuration of LD-pumped multimode graded-index fiber, which may generate at new wavelengths with a possibility of tuning in a broad range. The developed 2^nd^-order random Raman fiber laser may find applications as efficient/bright source for pumping various solid-state/fiber lasers including tandem pumping in high-power laser schemes used for material processing. High quality of the generated beam also offers an efficient frequency doubling that will transfer the generated spectrum into blue-green range (0.47–0.5 μm) thus enabling implementation of this source in bio-imaging and display technologies.
